# Supplementary material for: Deletion of wfs1 Impairs Oligodendrocyte Precursor Cells Dorsal Distribution and Myelination Through the wfs1-hmgcs1 Axis in Zebrafish
Source: Biology (Basel). 2026 Aug 16;15(16):1402. doi: 10.3390/biology15161402 (PMC13510151; doi:10.3390/biology15161402)
Supplement: Supplementary file 1 [file biology-15-01402-s001.zip › File S1-Transcriptome sequencing data and differentially expressed genes (DEGs) in wfs1-- zebrafish at 5 dpf/1.GO/wfs1KOvsWT/UP/wfs1KOvsWT.up_GObar.pdf]

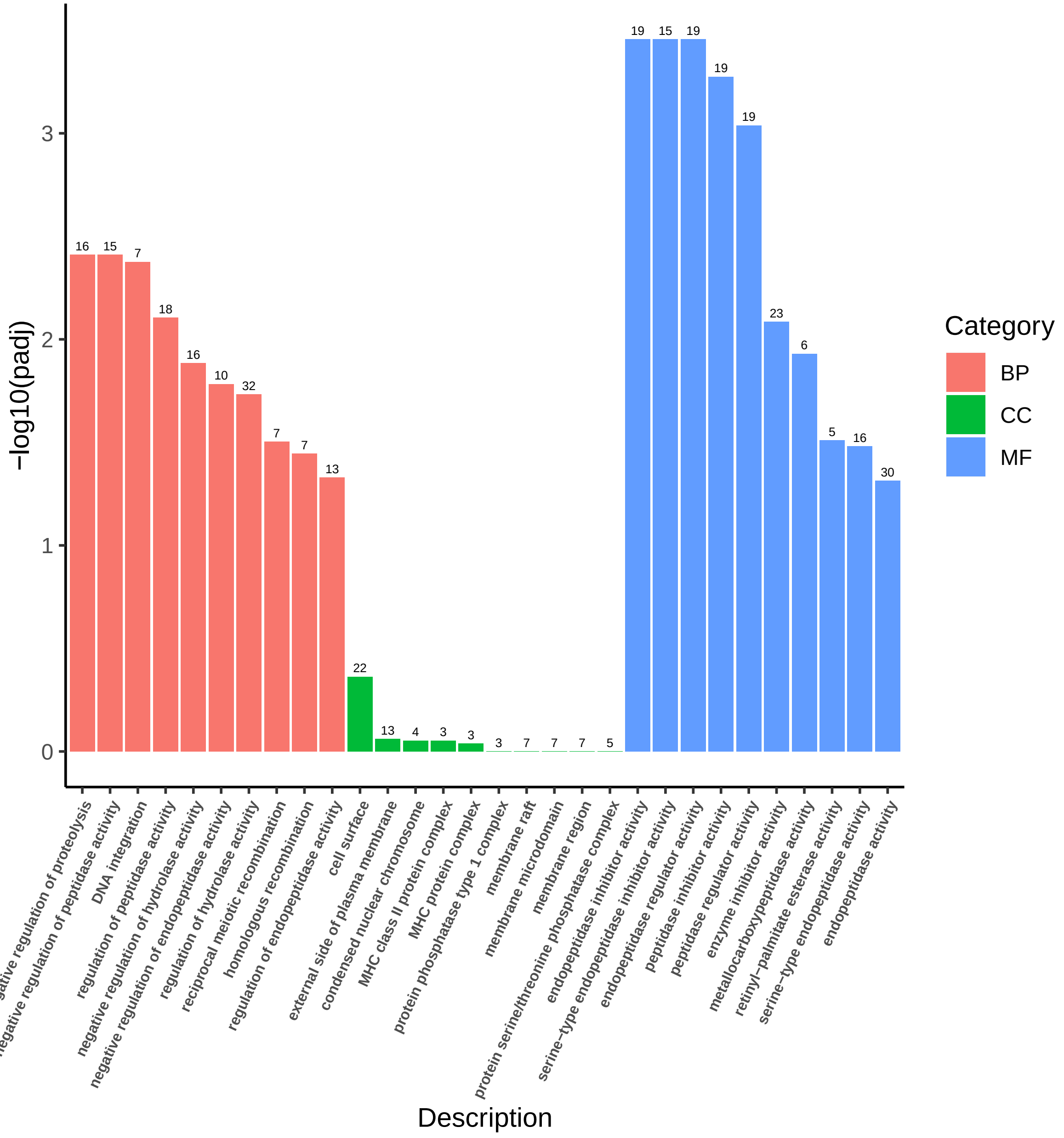

−log10(padj)
0
1
2
3
16
15
7
18
16
10
32
7
7
13
22
13
4
3
3
3
7
7
7
5
19
15
19
19
19
23
6
5
16
30
negative regulation of proteolysis
negative regulation of peptidase activity
DNA integration
regulation of peptidase activity
negative regulation of hydrolase activity
negative regulation of endopeptidase activity
regulation of hydrolase activity
reciprocal meiotic recombination
homologous recombination
regulation of endopeptidase activity
cell surface
external side of plasma membrane
condensed nuclear chromosome
MHC class II protein complex
MHC protein complex
protein phosphatase type 1 complex
membrane raft
membrane microdomain
membrane region
protein serine/threonine phosphatase complex
endopeptidase inhibitor activity
serine−type endopeptidase inhibitor activity
endopeptidase regulator activity
peptidase inhibitor activity
peptidase regulator activity
enzyme inhibitor activity
metallocarboxypeptidase activity
retinyl−palmitate esterase activity
serine−type endopeptidase activity
endopeptidase activity
Description
Category
BP
CC
MF
